# Reducing our environmental footprint and improving our health: greenhouse gas emission and land use of usual diet and mortality in EPIC-NL: a prospective cohort study

**DOI:** 10.1186/1476-069X-13-27

**Published:** 2014-04-07

**Authors:** Sander Biesbroek, H Bas Bueno-de-Mesquita, Petra HM Peeters, WM Monique Verschuren, Yvonne T van der Schouw, Gerard FH Kramer, Marcelo Tyszler, Elisabeth HM Temme

**Affiliations:** 1 Centre for Nutrition, Prevention and Health Services, The National Institute for Public Health and the Environment (RIVM), Antonie van Leeuwenhoek 9, Bilthoven 3721 MA, The Netherlands; 2 Department of Gastroenterology and Hepatology, University Medical Centre Utrecht, Heidelberglaan 100, 3508 GA Utrecht, The Netherlands; 3 School of Public Health, Imperial College London, London SW7 2AZ, United Kingdom; 4 Julius Centre, University Medical Centre Utrecht, Universiteitsweg 100, 3584 CG Utrecht, The Netherlands; 5 Blonk Consultants, Gravin Beatrixstraat 34, 2805 PJ Gouda, The Netherlands; 6 School of Business and Administration Sao Paolo, Av. Nove de Julho 2029 01313-902, São Paulo, Brasil

**Keywords:** Sustainability, Diet, Greenhouse gas emission, Land use, Health, Mortality, EPIC-NL, Prospective studies, Environmental impact, Substitution scenarios

## Abstract

**Background:**

Food choices influence health status, but also have a great impact on the environment. The production of animal-derived foods has a high environmental burden, whereas the burden of refined carbohydrates, vegetables and fruit is low. The aim of this study was to investigate the associations of greenhouse gas emission (GHGE) and land use of usual diet with mortality risk, and to estimate the effect of a modelled meat substitution scenario on health and the environment.

**Methods:**

The usual diet of 40011 subjects in the EPIC-NL cohort was assessed using a food frequency questionnaire. GHGE and land use of food products were based on life cycle analysis. Cox proportional hazard ratios (HR) were calculated to determine relative mortality risk. In the modelled meat-substitution scenario, one-third (35 gram) of the usual daily meat intake (105 gram) was substituted by other foods.

**Results:**

During a follow-up of 15.9 years, 2563 deaths were registered. GHGE and land use of the usual diet were not associated with all-cause or with cause-specific mortality. Highest vs. lowest quartile of GHGE and land use adjusted hazard ratios for all-cause mortality were respectively 1.00 (95% CI: 0.86-1.17) and 1.05 (95% CI: 0.89-1.23). Modelled substitution of 35 g/d of meat with vegetables, fruit-nuts-seeds, pasta-rice-couscous, or fish significantly increased survival rates (6-19%), reduced GHGE (4-11%), and land use (10-12%).

**Conclusions:**

There were no significant associations observed between dietary-derived GHGE and land use and mortality in this Dutch cohort. However, the scenario-study showed that substitution of meat with other major food groups was associated with a lower mortality risk and a reduced environmental burden. Especially when vegetables, fruit-nuts-seeds, fish, or pasta-rice-couscous replaced meat.

## Background

Current climate changes and the increased need for food underlines the importance of a sustainable food system [1]. Food choices influence health status, but also have a great impact on the environment through food production. Currently, the contribution of food production and consumption to the European Union’s total greenhouse gas emission (GHGE) is approximately 20-30% [2]. Individuals can directly influence this share via their own choice of food.

The largest environmental impact from food comes from the production and consumption of meat and dairy products; the estimated GHGE is 50% and use of total farmlands is 80% [3-5]. Meat and dairy products also supply about one-third of the dietary energy intake and are major sources of saturated fatty acids in the diet. Diets high in animal products and low in vegetables and fruits, like the Western diet, are associated with a higher risk of major chronic diseases [6-8]. A comparison between a meat-based and a lactoovovegetarian diet also showed that the typical Western diet is less sustainable [9].

A diet according to the Dutch national dietary guidelines would result in a 8% reduction in GHGE (men 13%, women 3%) when compared to the actual dietary pattern as observed in the Dutch national food consumption survey [3]. The dietary guidelines, established in 2006, promote a varied diet with plenty of fruit, vegetables and whole-grain cereal products [10]. A study from the UK modelled the health effects of three diets with a different carbon footprint reduction [11]. The results showed that a modelled 50% reduction of meat and dairy products iso-calorically replaced by vegetables, fruit, and cereals resulted in the highest GHGE reduction and most deaths delayed or averted per year. Another recent UK study investigated how consumer’s food choice could reduce food-derived GHGE [12]. Results showed a potential GHGE reduction of 35% if meat products were completely eliminated from the diet and 12% when avoidable food waste was cut out. Not eating foods grown in greenhouses or airfreighted to the UK could lead to another 5% reduction. Vieux et al. showed that when total caloric intake is reduced to meet the average individual’s energy needs, the diet-associated GHGE decreased between 2-11% [13]. In addition, when meat was iso-calorically substituted by fruit and vegetables either null or even positive diet-associated GHGE variations occurred. A modelled substitution of animal-derived food products by plant-derived food products resulted in substantial reductions of GHGE and land use [14,15] and a lower risk for all-cause mortality and coronary heart disease [16,17].

Although it is expected that a diet which is environmental friendlier also reduces mortality of chronic diseases, this has never been investigated in a real-life setting. Therefore, the aim of this study was to investigate to what extent an environmental friendlier diet is also a healthier diet by increasing survival rates in the EPIC-NL cohort study. The effects of food weight-based modelled substitution scenarios, in which part of the total meat intake was replaced by other food groups, on mortality and environmental impact were also investigated.

## Methods

### Study population

EPIC-NL is the Dutch contribution to European Prospective Investigation into Cancer and Nutrition [18]. EPIC-NL consists of 40 011 subjects of PROSPECT and MORGEN. The PROSPECT cohort included 17 357 women aged 49–70 years, participating in the national breast cancer screening program and living in the city of Utrecht and its surroundings [19]. The MORGEN cohort included 22 654 men and women aged 20–59 years, selected from random samples of the Dutch population in Amsterdam, Doetinchem, and Maastricht [20,21]. Both cohorts comply with the Declaration of Helsinki.

Exclusion criteria for the present analyses were no informed consent for follow-up of vital status (n = 956) and missing follow-up data (n = 16). Participants with a history of cancer (n = 1640), diabetes (n = 781), myocardial infarction (n = 536), stroke (n = 458), or a combination of these diseases (n = 249) were excluded because the usual diet reported by these persons may not reflect their diet before diagnosis. In addition, these participants have a higher risk of death. Subjects without dietary information (n = 154) were excluded. To exclude implausible values, participants in the highest and lowest 0.5% of the ratio of reported energy intake (based on the food frequency questionnaire (FFQ)) on energy requirement (estimated on the basis of basal metabolic rate (BMR)) were also excluded (n = 662). After these exclusions, 35 057 participants remained for the all-cause mortality survival analysis.

### Diet and environmental impact assessment

Usual daily dietary intake was estimated by a 178-item FFQ that has been validated against twelve 24-h recalls, and biomarkers in 24-h urine and blood [22,23]. Spearman rank correlation coefficients based on estimates of the FFQ and 24-h recalls were 0.51 for potatoes, 0.36 for vegetables, 0.68 for fruits, 0.39 for meat, 0.69 for dairy, 0.76 for sugar and sweet products, and 0.52 for biscuits and pastry in men. Results for women were similar.

Blonk Consultants assessed the environmental impact of the Dutch dietary habits [3]. To estimate sustainability scores, life cycle assessments (LCA) were performed for 254 food items. The LCA’s were cradle to grave and included production, processing, packaging, transport, storage, preparation, cooking, avoidable and unavoidable food waste (inedible parts) at home, and waste incineration. GHGE covers carbon dioxide (C0_2_) emissions through the use of fossil fuels, methane (CH_4_) released during the rearing of cattle and the cultivation of certain crops, and nitrous oxide (N_2_0) released from fertilizers, manure and ploughing of grassland [24,25]. GHGE is expressed as kg CO_2_-equivalents per day. Land use covers the surface needed for the production of food [24,25] and is expressed as m^2^*year per day. These LCA data were combined with the EPIC-NL FFQ data to calculate individual daily greenhouse gas emission and land use for each of our participants. The LCA scores were based on current production practices and assumed equal in the nineties when the FFQ was assessed.

### Participants characteristics

At baseline, study participants completed a questionnaire on the presence of chronic diseases and related potential risk factors, and medical and lifestyle factors [18]. Body mass index (BMI) was calculated by dividing weight by height squared. Educational level was coded in low (lower vocational training or primary school), medium (intermediate vocational training or secondary school), or high (higher vocational training or university). The smoking of cigarettes, pipe, or cigars was categorized as current, former, and never. Physical activity was assessed with the validated Cambridge Physical Activity Score (CPAI) [26].

### Mortality assessment

Vital status of all EPIC-NL participants was obtained through linkage with the municipal population registries. The information on vital status for the EPIC-NL cohort is complete until 11 April 2011 for MORGEN and until 4 July 2011 for PROSPECT. These data were retrieved from the GBA (Dutch Municipality Basic Administration).

Participants were followed for the occurrence of cancer, cardiovascular disease, respiratory disease and other causes by linkage to several disease registries (Dutch Cancer Registry and Dutch Hospital Discharge Diagnosis Database). Primary cause of death was coded according to the International Classification of Diseases (ICD). Incidence of cancer deaths was coded as 140–239 (ICD-9) or C00-D48 (ICD-10), incidence of cardiovascular disease (CVD) deaths as 390–459 (ICD-9) or I00-I99 (ICD-10), incidence of respiratory system disease mortality as 460–519 (ICD-9) or J00-J99 (ICD-10). The remaining causes of death were merged into the category ‘other causes’. Cause-specific mortality data were available until 31 December 2010. This is the most recent linkage to the database of Statistics Netherlands.

### Statistical analysis

Participants were followed over time until death from any cause, loss to follow-up, or were censored on 11 April 2011 for MORGEN and 4 July 2011 for PROSPECT. In the cause-specific mortality analysis, the censor date was 31 December 2010 for both cohorts.

Cox proportional hazard models were used to estimate crude and adjusted hazard ratios (HRs) with 95% confidence intervals (CI) for GHGE and land use in association with mortality. Using manual backward selection, covariates were excluded from the final model when the HR did not change ≥10% [27]. This manual selection was performed because no other prospective studies investigated the effect of the environmental impact of the diet, and therefore, there are no established confounders. The covariates BMI, educational level, smoking habits, physical activity, alcohol intake, and waist circumference were omitted from the final model whereas age and gender were retained. The covariate age failed to meet the proportional hazards assumption according to the Schoenfeld residuals test (p < 0.0001). Adjusted models were Cox stratified by age (continuous) to correct for this. To test for linear trends across categories, we modelled GHGE and land use by including the median value of each quartile as a continuous variable. By adding interaction terms to the model, we assessed deviation from multiplicative interaction for age, sex, BMI, smoking, and waist circumference. None of these factors modified the studied association. A test model in which quartiles of exposure were created from total GHGE and land use divided by total energy intake, GHGE/kJ and m^2^/kJ, showed very similar results (results not shown).

To study the effect of a modelled substitution of meat by other food components, both meat and the replacement component were added as continuous variables in the same multivariate model. Similar to previous studies, the difference in the parameter estimates and covariance was used to estimate HR and 95% CI [16,17]. The models were adjusted for major dietary and lifestyle factors (age, gender, BMI, smoking status, physical activity, energy intake, and alcohol intake). The investigated substitution component sources were potatoes, total vegetables, total fruit-nuts-seeds, pasta-rice-couscous, cheese, milk-based desserts, or fish. These food groups were selected because they can replace meat in a hot meal. In addition, they represent highly acceptable food products that are consumed in significant amounts in the current Dutch diet (Tables 1 and 2), and thus represent acceptable substitutions for meat. The modelled substitution was a one-third reduction (35-gram) of the average (105-gram, standard deviation of 55-gram) total daily meat intake in EPIC-NL. For realistic scenarios, we substituted by equal food weight and not the same amount of dietary energy. For example, in case of applying iso-caloric substitutions, an additional 300 gram of vegetables is needed to compensate for the energy intake of 35 gram of meat and this was assumed not to be realistic. Another argument for substitution based on food weight is that a large part of the adult Dutch population is overweight. This suggests that energy intake is high compared with energy requirements. Effects on environmental impact were based on the food group average GHGE and land use. The average environmental impact of meat was based on the proportional daily intake, i.e. non-processed meat accounts for 80% of total gram per day intake of meat.

**Table 1:** Contribution of different food groups to daily intake and environmental impact in EPIC-NL

**Food group**	**Gram/d (%)**	**C0**_**2**_**–eq (%)**	**Land use (%)**
Potatoes	3.5	1.9	1.2
Vegetables	4.4	5.5	3.6
Legumes	0.3	0.3	0.3
Fruit, nuts and seeds	6.9	5.6	4.4
Dairy			
Cheese	1.3	11.6	7.7
Milk^a^	9.4	9.5	6.5
Milk-based desserts^b^	3.5	4.1	2.6
Meat			
Non-processed meat^c^	2.5	25.7	28.1
Processed meat^d^	1.1	5.6	6.1
Cereals			
Bread products	5.0	3.4	4.8
Pasta, rice and couscous	1.6	1.5	2.6
Fish	0.4	2.1	0.8
Egg	0.5	1.2	1.8
Fat	0.9	2.3	5.0
Sugar and confectionary	1.5	2.5	1.7
Cake and biscuits	1.0	2.1	3.6
Beverages			
Non-alcoholic	48.0	9.4	10.9
Alcoholic	4.8	3.4	5.1
Condiments and sauces	0.7	0.8	1.2
Soups	2.4	0.6	0.2
Miscellaneous	0.3	2.1	2.0

^a^consists of milk, milk beverages (chocolate milk), and coffee milk; ^b^consists of (fruit)yoghurt, cream desserts, and milk-based puddings; ^c^non-processed meat: beef, pork, and chicken; ^d^processed meat: liver-containing items, ham, and miscellaneous types.

**Table 2:** Baseline characteristics by dietary greenhouse gas emission and land use in EPIC-NL

	**Greenhouse gas emission (CO**_**2**_**-eq/d)**		**Land use (m**^2^*year/d)	
**Characteristic**	**Quartile 1 <3.26**	**Quartile 4 >4.56**	**Quartile 1 <2.99**	**Quartile 4 >4.28**
No. of subjects	8770	8769	8769	8769
No. of deaths^a^	736 (8.4)	570 (6.5)	741 (8.5)	558 (6.4)
Person-years^b^	15.8 (14.6-17.0)	16.0 (14.7-17.2)	15.8 (14.6-16.9)	16.0 (14.7-17.2)
GHGE^b,c^	2.86 (2.56-3.07)	5.12 (4.79-5.62)	2.84 (2.55-3.14)	5.10 (4.71-5.62)
Land use^b, d^	2.62 (2.31-2.88)	4.78 (4.42-5.28)	2.61 (2.31-2.82)	4.80 (4.51-5.28)
Age (*years*)^b^	52 (44–59)	48 (37–54)	53 (44–60)	48 (37–54)
Male gender^a^	896 (10.2)	4521 (51.6)	766 (8.7)	4727 (53.9)
BMI (*kg*/*m*^*2*^)^b^	24.8 (22.4-27.1)	25.5 (23.2-28.0)	24.7 (22.3-27.0)	25.5 (23.2-28.0)
High education level^a,e^	1601 (18.4)	2025 (23.3)	1640 (18.8)	2141 (24.6)
Current smokers^a^	2466 (28.2)	3086 (35.3)	2179 (25.0)	2607 (29.8)
CPAI-‘active’^a,f^	3249 (37.1)	2488 (48.2)	3379 (38.5)	4070 (46.4)
Waist circumference (cm)^b^	81.0 (74.3-89.0)	87.3 (80.0-95.8)	81.0 (74.0-89.0)	87.8 (80.0-96.0)
Energy intake (M*J*)^b^	6.4 (5.6-7.3)	11.0 (9.4-12.8)	6.4 (5.6-7.4)	10.9 (9.43-12.8)
Ratio EI/BMR^b,g^	1.1 (1.0-1.3)	1.6 (1.4-1.9)	1.1 (1.0-1.3)	1.6 (1.4-1.9)
Alcohol use (*g*)^b^	2.1 (0.2-9.1)	10.3 (2.2-24.0)	1.5 (0.1-6.6)	12.9 (3.5-28.0)
Dietary intake^b^				
Potatoes	69 (41–105)	122 (75–179)	66 (41–101)	123 (76–180)
Vegetables	108 (82–140)	138 (107–175)	111 (84–145)	134 (105–171)
Legumes	5 (2–11)	8 (3–15)	5 (2–11)	8 (3–15)
Fruit, nuts & seeds	142 (92–250)	192 (118–300)	171 (109–262)	170 (104–274)
Dairy	261 (143–402)	533 (321–763)	308 (171–466)	453 (258–683)
Non-processed meat^h^	41 (23–58)	99 (84–125)	36 (21–51)	101 (87–126)
Processed meat^i^	15 (6–27)	40 (22–48)	14 (5–23)	43 (25–67)
Cereals	148 (11–193)	233 (171–311)	147 (111–191)	238 (174–315)
Fish	6 (2–14)	10 (-17)	7 (2–14)	9 (4–16)
Egg	11 (5–18)	16 (9–29)	11 (5–18)	17 (10–29)
Fat	20 (13–28)	34 (23–48)	19 (12–27)	36 (24–49)
Sugar & confectionary	31 (17–50)	48 (27–76)	31 (18–50)	47 (25–76)
Cake & biscuits	22 (11–37)	27 (14–45)	22 (11–37)	26 (13–44)
Beverages	1325 (1041–1670)	1717 (1368–2140)	1327 (1038–1678)	1726 (1395–2135)
Condiments & sauces	12 (5–22)	22 (11–33)	11 (5–22)	23 (12–34)
Soups	36 (17–72)	72 (33–107)	36 (17–72)	72 (33–107)
Miscellaneous	5 (2–11)	7 (3–15)	6 (2–11)	8 (4–15)

^a^Values displayed as frequency (percentage); ^b^Values displayed as median with interquartile range (25-75^th^ percentile); ^c^GHGE: greenhouse gas emission (C0_2_-eq/d); ^d^Land use (m^2^*year/d); ^e^college or university degree; ^f^Cambridge Physical Activity Score (inactive, moderately inactive, moderately active, active); ^g^Ratio of energy intake (EI) and basal metabolic rate (BMR); ^h^non-processed meat: beef, pork, and chicken; ^i^processed meat: liver-containing items, ham, and miscellaneous types.

All statistical analyses were performed using SAS software (version 9.3, SAS Institute Inc., Cary, NC, USA). A two-sided *p*-value of <0.05 was considered statistically significant.

## Results

During a median follow-up of 15.9 years, 2563 deaths were registered. The observed EPIC-NL cohort median value of GHGE was 3.87 kg CO_2_-equivalents/d and for land use 3.61 m^2^*year/d. While contributing 3.6% of daily intake weight (and 11% of daily energy intake), total meat intake accounts for approximately 30% of total dietary-derived GHGE and land use (Table 1). The impact of dairy and beverage consumption on the environment is substantial (dairy: 25% of GHGE and 17% of land use; beverages: 13% of GHGE and 16% of land use).

A higher energy, vegetables, fruits, dairy, meat, cereals, fat, soups, and alcohol intake, a lower age, an increased proportion of men, smokers, and higher activity level were associated with a higher environmental impact of usual diet. Educational level, waist to hip ratio, and body mass index (BMI) differed only slightly between the highest and lowest quartiles of GHGE and land use (Table 2).

In the crude Cox proportional hazards analyses, we observed an inverse association of total greenhouse gas emission of usual diet with all-cause mortality. The HR (95% confidence interval) of highest versus lowest quartile of GHGE was 0.76 (0.68-0.85) (Table 3). After multivariable adjustment, model 1, no association with risk was seen (HR of 1.00 (0.86-1.17)). Additional adjustment for energy intake, model 2, did not change the association. The findings from the fully adjusted model, all possible confounders included, were essentially similar to the sparsely adjusted model (model 1). Hazard ratios of highest versus lowest quartile of GHGE for adjusted cause-specific mortality models were for cancer 1.01 (0.86-1.34), CVD 0.90 (0.63-1.28), 1.12 (0.52-2.39) for respiratory diseases, and 0.91 (0.64-1.30) for other causes of death.

**Table 3:** Data for mortality risks according to greenhouse gas emissions of usual diet in EPIC-NL

	**Greenhouse gas emission (CO**_**2**_**-eq/d)**				***P *** **for linear trend**
	**<3.26**	**3.26 - 3.87**	**3.87 - 4.56**	**>4.56**	
**All-cause mortality**					
No. of participants	8770	8769	8771	8769	
No. of deaths	736	671	586	570	
Person-years, median	15.8	15.9	15.8	16.0	
Crude HR^a^ (95% CI)	1 (REF)	0.90 (0.81-1.00)	0.79 (0.71-0.88)	0.76 (0.68-0.85)	*P* < 0.0001^d^
Model 1^b^ HR	1	0.97 (0.84-1.12)	0.90 (0.77-1.05)	1.00 (0.86-1.17)	*P* = 0.7959
Model 2^b,c^ HR	1	0.96 (0.82-1.11)	0.87 (0.74-1.03)	0.95 (0.77-1.15)	*P* = 0.4266
**Cause-specific mortality**					
Cancer					
No. of deaths	327	324	274	268	
Crude HR^a^ (95% CI)	1 (REF)	0.99 (0.85-1.15)	0.83 (0.71-0.98)	0.81 (0.69-0.96)	*P* = 0.0031^d^
Model 1^b^ HR	1	1.01 (0.89-1.33)	0.93 (0.75-1.16)	1.01 (0.86-1.34)	*P* = 0.7654
CVD					
No. of deaths	164	146	115	120	
Crude HR^a^ (95% CI)	1 (REF)	0.89 (0.71-1.11)	0.70 (0.55-0.89)	0.73 (0.57-0.92)	*P* = 0.0023^d^
Model 1^b^ HR	1	0.92 (0.67-1.26)	0.83 (0.59-1.17)	0.90 (0.63-1.28)	*P* = 0.4681
Respiratory diseases					
No. of deaths	41	37	32	27	
Crude HR^a^ (95% CI)	1 (REF)	0.90 (0.58-1.40)	0.78 (0.79-1.23)	0.65 (0.40-1.06)	*P* = 0.0687
Model 1^b^ HR	1	1.01 (0.53-1.91)	0.76 (0.39-1.49)	1.12 (0.52-2.39)	*P* = 0.9945
Other causes					
No. of deaths	157	124	128	120	
Crude HR^a^ (95% CI)	1 (REF)	0.79 (0.62-0.99)	0.81 (0.64-1.02)	0.76 (0.60-0.96)	*P* = 0.0334^d^
Model 1^b^ HR	1	0.83 (0.59-1.15)	0.96 (0.68-1.35)	0.91 (0.64-1.30)	*P* = 0.7902

^a^HR: hazard ratio; ^b^Cox stratified for age (continuous) and adjusted for sex; ^c^Additional adjusted for energy intake.

^d^p value for linear trend significant (p < 0.05).

In crude analysis, total land use of usual diet was inversely associated with all-cause mortality (HR of highest versus lowest quartile: 0.74 (0.66-0.82)) (Table 4). However, after multivariable adjustment, we found a statistically non-significant HR of 1.05 (0.89-1.23). Correction for energy intake did not alter the association. Cause-specific adjusted HR’s were 1.10 (0.88-1.37) for cancer, 1.07 (0.75-1.54) for CVD, 1.19 (0.58-2.46) for respiratory diseases, and 0.88 (0.61-1.27) for deaths by remaining causes.

**Table 4:** Data for mortality risks according to total land use of usual diet in EPIC-NL

	**Land use (m**^2^*year/d)				***P *** **for linear trend**
	**<2.99**	**2.99 - 3.61**	**3.61 – 4.28**	**>4.28**	
**All-cause mortality**					
No. of participants	8769	8771	8770	8769	
No. of deaths	741	669	595	558	
Person-years, median	15.8	15.9	15.8	16.0	
Crude HR^a^ (95% CI)	1 (REF)	0.89 (0.80-0.99)	0.79 (0.71-0.88)	0.74 (0.66-0.82)	*P* < 0.0001^d^
Model 1^b^ HR	1	0.99 (0.86-1.15)	0.99 (0.85-1.14)	1.05 (0.89-1.23)	*P* = 0.6190
Model 2^b,c^ HR	1	0.99 (0.85-1.14)	0.97 (0.82-1.15)	1.03 (0.84-1.25)	*P* = 0.8534
**Cause-specific mortality**					
Cancer					
No. of deaths	326	317	282	268	
Crude HR^a^ (95% CI)	1 (REF)	0.97 (0.83-1.13)	0.86 (0.73-1.01)	0.82 (0.69-0.96)	*P* = 0.0057^d^
Model 1^b^ HR	1	1.05 (0.86-1.29)	0.99 (0.80-1.22)	1.10 (0.88-1.37)	*P* = 0.5291
CVD					
No. of deaths	164	151	112	118	
Crude HR^a^ (95% CI)	1 (REF)	0.91 (0.73-1.14)	0.68 (0.53-0.86)	0.71 (0.56-0.90)	*P* = 0.0010^d^
Model 1^b^ HR	1	1.03 (0.75-1.41)	0.97 (0.68-1.37)	1.07 (0.75-1.54)	*P* = 0.7666
Respiratory diseases					
No. of deaths	44	30	34	29	
Crude HR^a^ (95% CI)	1 (REF)	0.68 (0.42-1.07)	0.77 (0.49-1.20)	0.65 (0.41-1.04)	*P* = 0.1086
Model 1^b^ HR	1	0.81 (0.42-1.56)	0.97 (0.49-1.90)	1.19 (0.58-2.46)	*P* = 0.5950
Other causes					
No. of deaths	162	133	122	112	
Crude HR^a^ (95% CI)	1 (REF)	0.81 (0.65-1.02)	0.75 (0.59-0.95)	0.68 (0.54-0.87)	*P* = 0.0016^d^
Model 1^b^ HR	1	0.83 (0.60-1.16)	0.98 (0.70-1.36)	0.88 (0.61-1.27)	*P* = 0.6518

^a^HR: hazard ratio; ^b^Cox stratified for age (continuous) and adjusted for sex; ^c^Additional adjusted for energy intake.

^d^p value for linear trend significant (p < 0.05).

Modelling a substitution of 35 g/d of total meat intake by an equal amount of potatoes, pasta-rice-couscous, vegetables, fruit-nuts-seeds, milk-based desserts, fish, or cheese has environmental or health benefits (table 5). Reductions in total daily greenhouse gas emissions were 10.8% for potatoes, 10.1% for pasta-rice-couscous, 10.0% for vegetables, 10.0% for fruits-nuts-seeds, 10.0% for milk-based desserts, 4.5% for fish, 0.6% for cheese, and 11.5% for reducing meat intake by 35 gram without replacements based on the average carbon footprint of the usual diet in EPIC-NL. Reductions in land use were 11.3% for potatoes, 9.7% for pasta-rice-couscous, 10.8% for vegetables, and 10.3% for fruit-nuts-seeds, 10.9% for milk-based desserts, 9.8% for fish, 4.5% for cheese, and 11.7% without any replacement. In addition, favourable health effects of the substitutions were observed. When compared, 35 gram of pasta-rice-couscous instead of meat was associated with an 11% (95% CI, 4% to 16%) lower risk. A substitution by vegetables was associated with a 9% (95% CI, 3% to 15%) lower risk of all-cause mortality and by fruit-nuts-seeds with a 6% (95% CI, 1% to 10%) lower risk. A shift to 35 gram more milk-based dessert was associated with a borderline non-significant 4% (95% CI, 0% to 9%) lower risk. Substitution by fish was associated with a 19% (95% CI, 3% to 33%) lower risk. 35 gram more cheese instead of meat (HR: 6% (95% CI, -4% to 14%)) or potatoes (HR: 0% (95% CI, -6% to 7%)) was not associated with a lower all-cause mortality risk. Reducing intake of total meat by 35 gram without replacement was associated with a 4% (95% CI, 2% to 7%) lower mortality risk.

**Table 5:** Environmental impact of 35 gram modelled meat substitution by predefined food groups and all-cause mortality

**Substitute**	**Reduction GHGE (%)**^**a**^	**Reduction land use (%)**^**a**^	**Reduction mortality risk (%, 95% CI)**^**b**^
Potatoes	10.8	11.3	0 (-6 – 7)
Pasta-rice-couscous	10.1	9.7	11 (4 – 16)
Vegetables	10.0	10.8	9 (3 – 15)
Fruit, nuts and seeds	10.0	10.3	6 (1 – 10)
Milk-based desserts^c^	10.0	10.9	4 (0 – 9)
Fish	4.5	9.8	19 (3 – 33)
Cheese	0.6	4.5	6 (-4 – 14)
Remove 35 gram meat	11.5	11.7	4 (2 – 7)
(No replacement)			

^a^Based on the average greenhouse gas emission (GHGE) and land use in EPIC-NL; ^b^Cox stratified for age (continuous) and adjusted for gender, BMI (continuous), smoking status, physical activity, energy intake (continuous), and alcohol intake (continuous); c: consists of (fruit)yoghurt, cream desserts, and milk-based puddings.

## Discussion

In this large prospective cohort of Dutch men and women, we observed that the total environmental impact of usual diet was not associated with all-cause or cause-specific mortality. This indicates that an environmental friendlier diet is not necessarily a healthier diet. Even though meat only contributed for 3.6% to the total weight of daily intake in grams, it is responsible for approximately 30% of dietary greenhouse gas emission and land use. A 35 g/d reduction or shift from total meat intake to vegetables, fruit-nuts-seeds, pasta-rice-couscous, or fish would significantly increase survival rates (4-19%), reduce GHGE (4-12%), and land use (10-12%).

In this study, the environmental burden of the usual diet was divided into quartiles of total GHGE and land use to analyse the influence of diets with a higher impact on the relative risk for mortality. For this division no impact on mortality risk was observed in the Cox survival models. Other studies have suggested that a healthier diet may also be more sustainable [3,15]. A diet according to the Dutch Dietary Guidelines would result in 8% less GHGE and decrease land use by 21% compared to the average diet. However, a healthier diet and diet with a lower environmental impact do not necessarily need to be equally sustainable. For example, a healthy diet that includes fruits and vegetables with a high GHGE, rice instead of pasta or potatoes and more meat has twice the GHGE compared to an equally healthy low-GHGE diet [28]. On the other hand, a less healthy diet, with high quantities of sugars and refined carbohydrates, small quantities of meat, fruits and vegetables, can also have a low GHGE. Our modelled substitution scenario resulted in healthier diets with reduced environmental impact. Substitutions of meat lead to a double benefit in both health and reduced environmental impact aspects. However, a healthier diet is not necessarily accompanied by a lower GHGE or less land use.

The Dutch diet is relatively high in animal-derived products and refined carbohydrates and low in fruit and vegetables. Within the dietary range of this cohort, there was no significant association between the overall daily GHGE and land use and mortality. Although total GHGE and land use were not associated with mortality, modelling a one-third reduction of total meat, a major contributor to dietary GHGE and land use, resulted in both reduced mortality risk as well as reduced environmental impact. The 35-gram reduction of meat was well within the intake variation (standard deviation) of 55 gram and is thus a realistic scenario. Meat intake has been linked to an increased risk of mortality before [29]. In addition, other meat substitution studies reported reduced mortality [17] or cardiovascular risks [16]. Temme *et al.* showed that a complete replacement of meat and dairy by a variety of plant-derived foods would not affect total iron intake, reduce saturated fatty acid intake, and reduce land use by around 50% in Dutch female young adults [30].

Substituting high-GHGE with low-GHGE meats could also contribute to increased survival rates and reduced environmental impact. Replacing red meat with poultry would reduce the environmental impact (data Blonk Consultants ) and is associated with reduced mortality risk [17]. In addition, processed meat intake appears to be stronger associated with several morbidity outcomes than red meat [31]. Replacement of meat by fish can be considered controversial from an ecological point of view, because of sustainability concerns of the current ocean fishing and fish cultivation practices.

A New Zealand study presented findings of scenario development with linear programming that determined several dietary patterns to cover nutrient intake at low cost and low GHGE profiles [32]. The study suggests that these results could provide guidance to governments decisions around the focus of their food policies, i.e. food taxes, healthy food vouchers and subsidies. An UK study investigated the effect of incorporating the societal cost of GHGE into the price of foods [33]. A scenario in which a higher taxation rate is calculated for foods above GHGE average shows that this could save 7770 lives in the UK each year, reduce GHGE and generate tax revenue. These studies highlight the potential benefits of such policy measures on health and environment impact of the diet.

Our study has some strengths and limitations. The combination of sustainability of the usual diet and health was not previously studied in a large prospective cohort with a follow-up time of 16 years. The participants of this cohort were sampled from four different geographic areas in the Netherlands and therefore the results may be extrapolated to the Dutch population. In addition, mean GHGE and land use in our cohort were similar to the Dutch Consumption Survey of 1998 [3]. The dietary assessment took place only in the nineties, while nowadays people might have different eating patterns and eat foods that are produced differently. A FFQ is designed to rank people according to their diet. Therefore, the modelled substitution of the 35 g/d of meat was not based on actual intake but was estimated with usual intake. However, our outcomes clearly demonstrate health and environmental benefits from a dietary shift towards lower meat consumption.

The scope of this study is limited to substitutions of an equivalent quantity in grams. Future research may include iso-caloric substitutions or nutritional component equivalency of meat substitutions. In addition, within food groups the environmental impact can vary per product due to farming methods, animal feed, use of side products, transport, and growing conditions [24]. Taking the variety of distributions of environmental impact for every stage of the production process would allow for variance estimation of the environmental impact of a food group. This would further improve the GHGE and land use estimates used in our study. Other research may focus on the role of governmental decisions on consumer behaviour and its efficacy. Examples of governmental actions could be a food-labelling system that indicates GHGE per 100-gram product, food taxes based on a combination of health aspects and environmental impact of a product, or media campaigns to inform consumers of environmental impact of foods.

## Conclusions

The Dutch diet is relatively high in animal-derived food products and refined carbohydrates and low in fruit and vegetables. Within the dietary range of this population-based cohort, there were no significant associations between overall daily dietary-derived GHGE and land use and mortality. However, a modelled reduction of 35 gram meat which was replaced with vegetables, fruits, fish, or cereal-rice-couscous resulted in lower GHGE and land use as well as decreased all-cause mortality risk. The results of our study emphasise that a healthier diet is not necessarily a more sustainable diet, and the other way around. Nevertheless, a reduction of meat consumption can influence both health and environmental aspects.

## Abbreviations

BMI: Body mass index; CPAI: Cambridge physical activity index; EPIC: European Prospective Investigation into Cancer and Nutrition; FFQ: Food frequency questionnaire; GHGE: Greenhouse gas emission; HR: Hazard ratio; 95% CI: 95% confidence interval.

## Competing interests

The authors declare that they have no competing interests.

## Authors’ contributions

SB carried out the statistical analysis, prepared the tables and figures, and wrote the paper, taking into account comments from all the co-authors. EHMT and HBB-d-M initiated and designed this study. EHMT was the overall project coordinator. HBB-d-M and EHMT were members of the writing group and gave input on the statistical analysis and interpretation of the results. HBB-d-M, PHMP, WMMV, and YTS are members of the EPIC-NL steering committee. All authors provided comments and suggestions on the manuscript and approved the final version.

## References

[B1] Lang T Barling D Nutrition and sustainability: an emerging food policy discourse Proc Nutr Soc 2013 72 1 12 10.1017/S002966511200290X 23217475

[B2] Tukker A Huppes G Guinée J Heijungs R Koning A Oers L Suh S Geerken T Holderbeke M Jansen B Environmental Impact of Products (EIPRO) Analysis of the life cycle environmental impacts related to the final consumption of the EU-25 2006 Brussels: European Commision, Joint Research Centre, Institure for Prospective Technological Studies

[B3] Marinussen M Kramer G Pluimers J Blonk H The environmental impact of our diet - an analysis based on de nutitional consumption survey of 2007–2010 (in Dutch, summary in English) Book The environmental impact of our diet - an analysis based on de nutitional consumption survey of 2007–2010 (in Dutch, summary in English) 2012 Gouda: Blonk Milieu Advies

[B4] Godfray HCJ Beddington JR Crute IR Haddad L Lawrence D Muir JF Pretty J Robinson S Thomas SM Toulmin C Food security: the challenge of feeding 9 billion people Science 2010 327 812 818 10.1126/science.1185383 20110467

[B5] Westhoek H Rood T Van den Berg M Janse J Nijdam D Reudink M Stehfest E The protein puzzle 2011 The Hague: PBL Netherlands Environmental Assessment Agency 221

[B6] Lock K Pomerleau J Causer L Altmann DR McKee M The global burden of disease attributable to low consumption of fruit and vegetables: implications for the global strategy on diet Bull World Health Organ 2005 83 100 108 15744402 PMC2623811

[B7] Sinha R Cross AJ Graubard BI Leitzmann MF Schatzkin A Meat intake and mortality: a prospective study of over half a million people Arch Intern Med 2009 169 562 571 10.1001/archinternmed.2009.6 19307518 PMC2803089

[B8] Cordain L Eaton SB Sebastian A Mann N Lindeberg S Watkins BA O’Keefe JH Brand-Miller J Origins and evolution of the Western diet: health implications for the 21st century Am J Clin Nutr 2005 81 341 354 15699220 10.1093/ajcn.81.2.341

[B9] Pimentel D Pimentel M Sustainability of meat-based and plant-based diets and the environment Am J Clin Nutr 2003 78 660S 663S 12936963 10.1093/ajcn/78.3.660S

[B10] Health Council of the Netherlands Guidelines for a healthy diet 2006 2006 The Hague: Health Council of the Netherlands publciation no. 2006/21

[B11] Scarborough P Allender S Clarke D Wickramasinghe K Rayner M Modelling the health impact of environmentally sustainable dietary scenarios in the UK Eur J Clin Nutr 2012 66 6 710 715 10.1038/ejcn.2012.34 22491494 PMC3389618

[B12] Hoolohan C Berners-Lee M McKinstry-West J Hewitt C Mitigating the greenhouse gas emissions embodied in food through realistic consumer choices Energy Policy 2013 63 1065 1074

[B13] Vieux F Darmon N Touazi D Soler L Greenhouse gas emissions of self-selected individual diets in France: Changing the diet structure or consuming less? Ecol Econ 2012 75 91 101

[B14] Stehfest E Bouwman L van Vuuren DP den Elzen MGJ Eickhout B Kabat P Climate benefits of changing diet Clim Chang 2009 95 83 102 10.1007/s10584-008-9534-6

[B15] Macdiarmid JI Kyle J Horgan GW Loe J Fyfe C Johnstone A McNeill G Sustainable diets for the future: can we contribute to reducing greenhouse gas emissions by eating a healthy diet? Am J Clin Nutr 2012 96 632 639 10.3945/ajcn.112.038729 22854399

[B16] Bernstein AM Sun Q Hu FB Stampfer MJ Manson JE Willett WC Major dietary protein sources and risk of coronary heart disease in women Circulation 2010 122 876 883 10.1161/CIRCULATIONAHA.109.915165 20713902 PMC2946797

[B17] Pan A Sun Q Bernstein AM Schulze MB Manson JE Stampfer MJ Willett WC Hu FB Red meat consumption and mortality: results from 2 prospective cohort studies Arch Intern Med 2012 172 555 10.1001/archinternmed.2011.2287 22412075 PMC3712342

[B18] Beulens JWJ Monninkhof EM Verschuren WMM van der Schouw YT Smit J Ocke MC Jansen EHJM van Dieren S Grobbee DE Peeters PHM Cohort profile: the EPIC-NL study Int J Epidemiol 2010 39 1170 1178 10.1093/ije/dyp217 19483199

[B19] Boker LK Van Noord P Van Der Schouw Y Koot N de Mesquita HBB Riboli E Grobbee D Peeters P Prospect-EPIC Utrecht: study design and characteristics of the cohort population Eur J Epidemiol 2001 17 1047 1053 10.1023/A:1020009325797 12380720

[B20] Verschuren W Blokstra A Picavet H Smit H Cohort profile: the Doetinchem cohort study Int J Epidemiol 2008 37 1236 1241 10.1093/ije/dym292 18238821

[B21] Blokstra A Smit H Bueno de Mesquita H Seidell J Verschuren W Monitoring of risk factors and health in the Netherlands (MORGEN-cohort) 1993–1997. Lifestyle- and risk factors: prevalences and trends (in Dutch) 2005 Bilthoven: RIVM

[B22] Ocke MC Bueno-de-Mesquita HB Goddijn HE Jansen A Pols MA van Staveren WA Kromhout D The Dutch EPIC food frequency questionnaire. I. Description of the questionnaire, and relative validity and reproducibility for food groups Int J Epidemiol 1997 26 S37 10.1093/ije/26.suppl_1.S37 9126532

[B23] Ocke MC Bueno-de-Mesquita HB Pols MA Smit HA van Staveren WA Kromhout D The Dutch EPIC food frequency questionnaire. II Relative validity and reproducibility for nutrients International J Epidemiol 1997 26 S49 10.1093/ije/26.suppl_1.s49 9126533

[B24] Garnett T Cooking up a storm: Food, greenhouse gas emissions and our changing climate Book Cooking up a storm: Food, greenhouse gas emissions and our changing climate 2008 Food Climate Research Network, Centre for Environmental Strategy, University of Surrey

[B25] IPCC Climate change 2007: The physical science basis 2007 Geneva: Cambrigde University Press

[B26] Pols MA Peeters P Ocke MC Slimani N Bueno-de-Mesquita HB Collette H Estimation of reproducibility and relative validity of the questions included in the EPIC Physical Activity Questionnaire Int J Epidemiol 1997 26 S181 10.1093/ije/26.suppl_1.S181 9126546

[B27] Petrie A Sabin C Medical statistics at a glance 2009 Oxford: Blackwell Pub

[B28] Macdiarmid J Is a healthy diet an enviromental sustainable diet? Proc Nutr Soc 2013 72 13 20 10.1017/S0029665112002893 23186839

[B29] Rohrmann S Overvad K Bueno-de-Mesquita HB Jakobsen MU Egeberg R Tjønneland A Nailler L Boutron-Ruault M-C Clavel-Chapelon F Krogh V Meat consumption and mortality-results from the European Prospective Investigation into Cancer and Nutrition BMC Med 2013 11 63 10.1186/1741-7015-11-63 23497300 PMC3599112

[B30] Temme E van der Voet H Thissen J Verkaik-Kloosterman J van Donkersgoed G Nonhebel S Replacement of meat and dairy by plant-derived foods: estimated effects on land use, iron and SFA intakes in young Dutch adult females Public Health Nutr 2013 16 10 1900 1907 10.1017/S1368980013000232 23425363 PMC10271561

[B31] Micha R Wallace SK Mozaffarian D Red and processed meat consumption and risk of incident coronary heart disease, stroke, and diabetes mellitus a systematic review and meta-analysis Circulation 2010 121 2271 2283 10.1161/CIRCULATIONAHA.109.924977 20479151 PMC2885952

[B32] Wilson N Nghiem N Mhurchu CN Eyles H Baker MG Blakely T Foods and dietary patterns that are healthy, low-cost, and environmentally sustainable: a case study of optimization modeling for New Zealand PLoS One 2013 121 21 2271 2283 10.1371/journal.pone.0059648 PMC3609827 23544082

[B33] Briggs AD Kehlbacher A Tiffin R Garnett T Rayner M Scarborough P Assessing the impact on chronic disease of incorporating the societal cost of greenhouse gases into the price of food: an econometric and comparative risk assessment modelling study BMJ Open 2013 3 10 e003543 24154517 10.1136/bmjopen-2013-003543 PMC3808835

